# Editorial: PCSK9: Importance in Physiology and Pathophysiology

**DOI:** 10.3389/fphys.2021.706115

**Published:** 2021-07-26

**Authors:** Rainer Schulz, Ioanna Andreadou, Péter Ferdinandy

**Affiliations:** ^1^Institute of Physiology, Justus Liebig University Giessen, Giessen, Germany; ^2^Laboratory of Pharmacology, Faculty of Pharmacy, National and Kapodistrian University of Athens, Athens, Greece; ^3^Department of Pharmacology and Pharmacotherapy, Semmelweis University, Budapest, Hungary; ^4^Pharmahungary Group, Szeged, Hungary

**Keywords:** proprotein convertase subtilisin kexin type 9, LDL - cholesterol, heart, skelelal muscle, therapy

Proprotein Convertase Subtilisin/Kexin type 9 (PCSK9) is involved in cholesterol homeostasis. After binding to the complex low-density lipoprotein (LDL)-receptor, PCSK9 induces its intracellular degradation, thereby reducing serum LDL clearance leading to increased circulating LDL concentrations which contributes to cardiovascular disease development and progression. Therefore, circulating PCSK9 has become a novel drug target in lipid-lowering therapy as reviewed by Katzmann et al. Recent clinical trials, however, show that PCSK9 inhibitors can also effectively reduce plasma lipoprotein(a) (Lp(a))concentration, which—when increased—shows a strong association with increased risks of coronary heart disease and cardiovascular disease as demonstrated in epidemiological, genome-wide association, and Mendelian randomization studies. The mode of action on how PCSK9 inhibitors affect Lp(a) depends on co-treatments and has been reviewed by Ying et al..

PCSK9 is almost ubiquitously expressed in different cell types throughout the body and cellular expression levels are affected by age, sex (Kucsera et al.), and comorbidities (example such as hypertension, see Wolf et al.) and circulating PCSK9 levels are positively correlated to these confounders in patients.

Although PCSK9 is now been recognized as a ubiquitously expressed modifier of cellular function and signaling molecules, its physiological role in different organs such as the brain, skeletal muscle, kidney, and the intestine is not well-understood and our current knowledge of its function in these organs is reviewed in detail by Schlüter et al.. In the skeletal muscles of rats, the expression of PCSK9 is lower in the soleus as compared to the gastrocnemius muscle. As the soleus muscle is using preferentially glucose as substrate whereas the gastrocnemius muscle prefers fatty acids this finding might point to a role for PCSK9 in the regulation of skeletal muscle metabolism. Interestingly, exercise increased the expression of PCSK9 in both muscles in normotensive but not in hypertensive rats, again pointing to the importance of confounders in PCSK9 regulation in different organs (Wolf et al.s).

In the heart and especially in cardiomyocytes, PCSK9 is up-regulated and released during ischemia and decreasing extracellular PCSK9 expression impacts on infarct size and cardiac dysfunction (as reviewed by Andreadou et al.; Schlüter et al.). Clinical data support the notion that inhibition of extracellular PCSK9 is associated with a reduction in the incidence of myocardial infarction and coronary revascularization in subjects with increased cardiovascular risk (Katzmann et al.). The mechanisms by which extracellular PCSK9 impact on cardiomyocytes are incompletely understood but involve receptors other than the LDL-receptor which are affected by PCSK9 such as the LDL receptor-related protein 1 (LRP1). Treatment of adult rat cardiomyocytes with recombinant PCSK9 but not knockdown of endogenous PCSK9 results in a strong reduction in LRP1 protein expression and as a consequence the activation of protein kinases, the mitochondrial biogenic effect and the impact on glucose metabolism induced by adipocytokines are blunted (Rohrbach et al.). Thus, PCSK9 affects intercellular communication (from adipocytes toward cardiomyocytes) but, importantly, under stress conditions, signals from organs such as the kidney and the heart affect hepatic PCSK9 expression and thereby the plasma concentration of PCSK9 (Schlüter et al.). Similarly, disease states such as non-alcoholic steatohepatitis impair local PCSK9 expression and the potential of the liver to take up cholesterol a process showing sex differences (Kucsera et al.). Previous studies in PCSK9 knock-out mice on a high-fat diet demonstrated more excessive hepatocyte lipid droplet formation, augmented inflammatory gene expression, and more pronounced liver fibrosis, suggesting that PCSK9 deficiency could promote hepatic steatosis at least in preclinical models. Whether the pharmacological inhibition of PCSK9 either by monoclonal antibodies or silencing RNAs has similar effects in morbidly obese patients with non-alcoholic fatty liver disease or non-alcoholic steatohepatitis has not been studied yet.

Taken together, PCSK9 not only modifies LDL receptor cycling in the liver thereby affecting circulating LDL concentration. Rather, a complex interplay of different organs and the local (autocrine) and systemic (endocrine) effects of PCSK9 exist (Figure 1).

**Figure 1:**
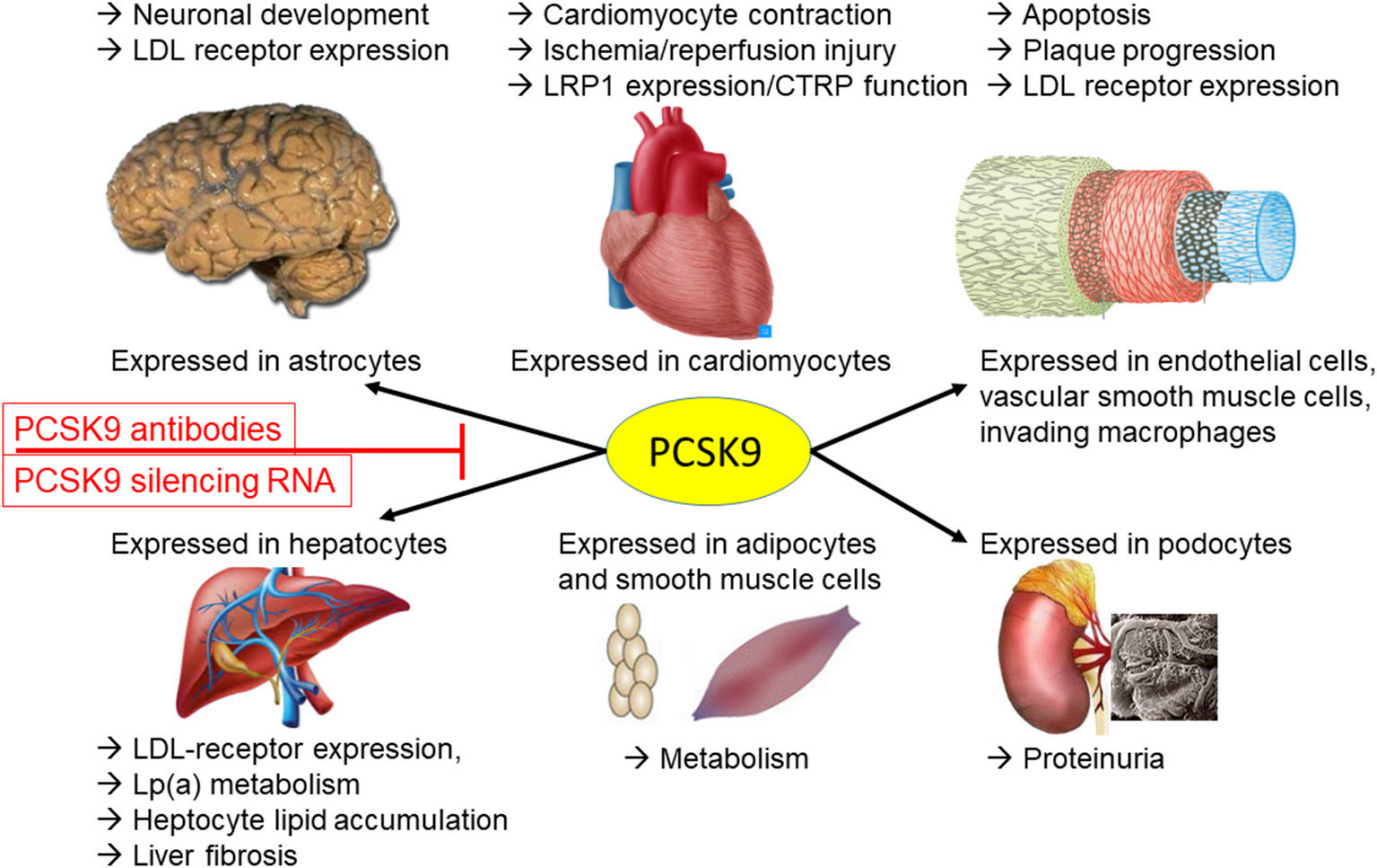
PCSK9 is expressed in many cell types throughout the body. Here, PCSK9 can be released under certain pathophysiological conditions and/or comorbidities and either act locally or contribute to organ-organ interaction by increasing the circulating PCSK9 concentration. LDL, low-density lipoprotein; Lp(a), lipoprotein a; CTRP, C1q/TNF-related proteins; LRP1, low density lipoprotein receptor-related protein 1.

## Author Contributions

All authors listed have made a substantial, direct and intellectual contribution to the work, and approved it for publication.

## Conflict of Interest

PF is the founder and CEO of Pharmahungary, a group of R&D companies. The remaining authors declare that the research was conducted in the absence of any commercial or financial relationships that could be construed as a potential conflict of interest.

## Publisher's Note

All claims expressed in this article are solely those of the authors and do not necessarily represent those of their affiliated organizations, or those of the publisher, the editors and the reviewers. Any product that may be evaluated in this article, or claim that may be made by its manufacturer, is not guaranteed or endorsed by the publisher.

